# Mechanisms and Evolution of Antimicrobial Resistance in Ophthalmology: Surveillance, Clinical Implications, and Future Therapies

**DOI:** 10.3390/antibiotics14111167

**Published:** 2025-11-20

**Authors:** Isaiah Osei Duah Junior, Josephine Ampong, Cynthia Amaning Danquah

**Affiliations:** 1 Department of Optometry and Visual Science, College of Science, Kwame Nkrumah University of Science and Technology, PMB, Kumasi, Ghana or oseiduahisaiah@gmail.com (I.O.D.J.); jampong2@st.knust.edu.gh (J.A.); 2 Department of Biological Sciences, College of Science, Purdue University, West-Lafayette, IN 47906, USA; 3 Department of Psychology, College of Health and Human Science, North Carolina Agricultural and Technical State University, Greensboro, NC 27403, USA; 4 Department of Pharmacology, Faculty of Pharmacy and Pharmaceutical Sciences, College of Health Sciences, Kwame Nkrumah University of Science and Technology, PMB, Kumasi, Ghana

**Keywords:** antibiotic resistance, ocular infections, multidrug-resistant pathogens, diagnostics, antimicrobial stewardship

## Abstract

Antimicrobial resistance (AMR) is a growing global health concern with profound implications for ophthalmology, where it compromises the management of ocular infections such as bacterial keratitis, conjunctivitis, endophthalmitis, and postoperative complications. Resistance in common ocular pathogens, including *Staphylococcus aureus* (*S. aureus*), *Streptococcus pneumoniae* (*S. pneumoniae*), *Pseudomonas aeruginosa* (*P. aeruginosa*), and coagulase-negative staphylococci (CoNS) emerge through genetic mutations, horizontal gene transfer, and biochemical mechanisms such as enzymatic degradation, target modification, efflux pumps, and reduced membrane permeability. Biofilm formation further complicates eradication on the ocular surface and interior. The key drivers of resistance include inappropriate or prolonged topical antibiotic use, routine prophylaxis in ocular surgery, subtherapeutic dosing, and cross-resistance with systemic antimicrobials. The rise in multidrug-resistant strains, particularly methicillin-resistant *S. aureus*, fluoroquinolone-resistant *P. aeruginosa*, and drug-resistant *S. pneumoniae* has been linked to delayed treatment response, increased healthcare costs, and sight-threatening outcomes. Recent advances in rapid diagnostics, molecular assays, and point-of-care testing support earlier and more precise detection of resistance, enabling timely therapeutic decisions. Promising strategies to address AMR in ophthalmology include antimicrobial stewardship, novel drug delivery platforms, and alternative approaches such as bacteriophage therapy and antimicrobial peptides. Emerging tools, including genomic surveillance, artificial intelligence (AI)-driven resistance prediction, and personalized antimicrobial regimens, further expand opportunities for innovation. Collectively, this review synthesizes current evidence on AMR in ocular disease, summarizing patterns of resistance, underlying mechanisms, and clinical consequences, while highlighting strategies for mitigation and underscoring the need for global awareness and collaboration among clinicians, researchers, and policymakers to safeguard vision.

## 1. Introduction

Antimicrobial resistance represents one of the most pressing global health challenges of the 21st century [1,2]. The World Health Organization (WHO) has repeatedly emphasized the threat posed by resistant pathogens, projecting that, if unmitigated, AMR could cause up to 10 million deaths annually by 2050 and impose substantial economic burdens on healthcare systems worldwide [1,2]. While much attention has focused on systemic infections, AMR in ophthalmology is an emerging concern with significant clinical implications [3,4]. The eye is uniquely vulnerable to infections due to its exposed surface, rich vascular supply, and specialized immune defenses [5]. Ocular infections, including bacterial keratitis, conjunctivitis, endophthalmitis, and postoperative infections, can rapidly progress and threaten vision, making timely and effective antimicrobial therapy essential [5]. However, the growing prevalence of resistant ocular pathogens threatens to undermine the efficacy of standard treatments, prolong recovery, and increase the risk of vision loss [6].

The resistance to ocular pathogens is driven by multiple interrelated factors [4,7]. Overuse and misuse of topical antibiotics, prophylactic administration in ocular surgery, subtherapeutic dosing, and poor patient adherence all contribute to selective pressure favoring resistant strains [4,7]. Additionally, cross-resistance with systemic antibiotics further complicates management, as commonly used drugs lose efficacy against both ocular and systemic infections [4,8]. The clinical consequences are significant: multidrug-resistant infections are associated with delayed healing, higher rates of complications, increased healthcare costs, and, in severe cases, permanent visual impairment [4,8,9,10]. Pathogens such as methicillin-resistant *S. aureus* (MRSA), *P. aeruginosa*, and drug-resistant *S. pneumoniae* have emerged as major challenges in ophthalmic practice, highlighting the urgent need for comprehensive strategies to detect, monitor, and manage resistance [11,12,13,14]. Understanding the epidemiology, mechanisms, and drivers of AMR in ocular pathogens is crucial to inform clinical decision-making and guide public health interventions. Advances in molecular diagnostics, rapid point-of-care testing, and genomic surveillance provide opportunities to detect resistance more efficiently and to tailor therapy to the individual patient [15,16,17]. Moreover, evolving strategies, ranging from antimicrobial stewardship (AMS) programs to novel drug delivery systems and alternative therapies such as phage therapy, offer promise for mitigating resistance and improving outcomes [18,19,20].

To this end, the aim of this review is to provide a comprehensive synthesis of the current evidence on AMR in ophthalmology. Bibliographic searches were conducted over a three-month period, from inception to date, across multiple standard databases, including PubMed, Scopus, Web of Science, MEDLINE, Embase, and Google Scholar. Only published, peer-reviewed articles in the English language were systematically synthesized. In particular, the review employed a narrative synthesis framework to consolidate patterns of resistance in common ocular pathogens, explore the genetic and biochemical mechanisms underlying resistance, evaluate the clinical impact on patient care, and discuss strategies to mitigate resistance. Additionally, it highlights emerging diagnostic tools, innovative therapeutic approaches, and future directions, including the application of AI and the importance of global collaboration. By integrating epidemiological, mechanistic, and clinical perspectives, this review aims to inform both clinical practice and research, ultimately seeking to preserve the efficacy of antimicrobial therapies and safeguard visual health in the face of an evolving resistance landscape.

## 2. Global and Regional Surveillance of Antimicrobial Resistance in Ophthalmology

Ocular infections such as conjunctivitis, keratitis, and endophthalmitis are often treated empirically, but rising AMR reduces treatment efficacy, increases costs, and threatens vision. Understanding pathogen- and region-specific resistance patterns is critical to mitigating AMR and optimizing patient visual outcomes.

Bacterial keratitis is a potentially sight-threatening condition, most frequently caused by *S. aureus*, *S. pneumoniae*, *P. aeruginosa*, and CoNS [11,21]. Among these, methicillin-resistant *S. aureus* (MRSA) has emerged as a major concern, accounting for approximately 20–60% of keratitis cases globally, with particularly high prevalence reported in North America and Asia [11,22,23]. Similarly, *P. aeruginosa*, a leading cause of contact lens–associated keratitis, has shown increasing resistance to fluoroquinolones, thereby complicating empirical first-line therapy [24]. Although CoNS are generally less virulent, they are important pathogens in post-surgical keratitis and endophthalmitis, with methicillin resistance rates exceeding 40% in certain regions [24,25]. Conjunctivitis, another common ocular infection, is typically caused by *S. aureus*, *Streptococcus* species, and *Haemophilus influenzae*. However, growing resistance to macrolides, aminoglycosides, and tetracyclines, especially in pediatric populations where macrolides are widely prescribed empirically, has become an increasing clinical challenge [3,6]. Endophthalmitis, though less common, carries a significant risk of vision loss. Post-surgical cases are most often attributed to CoNS and *Streptococcus* species [26,27,28], and recent studies indicate rising methicillin and fluoroquinolone resistance among these isolates, particularly in nosocomial settings [29,30]. Moreover, Gram-negative organisms such as *Pseudomonas* and *Enterobacter species* are exhibiting increasing rates of multidrug resistance (MDR), further limiting therapeutic options for ocular infections [31].

Of note, the Global Antimicrobial Resistance and Use Surveillance System (GLASS), launched in 2015, is the WHO’s flagship program for monitoring AMR worldwide [32]. It collects standardized data on priority pathogens from participating countries, including *S. aureus*, *P. aeruginosa*, and *S. pneumoniae*, which are relevant to ocular infections. GLASS data helps identify emerging resistance trends, track MDR strains, and guide empirical treatment policies [33]. While GLASS primarily reports on systemic infections, its pathogen-focused data can be extrapolated to ophthalmology, particularly for bloodstream-associated or surgical infections that may affect the eye [34] as shown in Table 1.

In North America, surveillance programs such as ARMOR (Antibiotic Resistance Monitoring in Ocular Microorganisms) provide long-term insights into AMR trends [12,35,36]. The ARMOR is a specialized surveillance program designed to track AMR in ocular pathogens [12,35,36]. It collects isolates, mainly of MRSA, fluoroquinolone-resistant *P. aeruginosa*, and penicillin-resistant *S. pneumoniae*, from patients with conjunctivitis, keratitis, and endophthalmitis, analyzing susceptibility to commonly used topical and systemic antibiotics [36]. Together, this program provides ophthalmologists with up-to-date resistance profiles, aiding empirical therapy decisions and stewardship practices [36].

Ocular TRUST (Tracking Resistance in the United States Today) is a multicenter program in the United States that monitors resistance among ocular pathogens [37]. It evaluates susceptibility patterns for *S. aureus*, *S. pneumoniae*, *Haemophilus influenzae*, and *P. aeruginosa* isolates from ocular infections [37]. Ocular TRUST has documented the rise in MRSA in conjunctivitis and keratitis and the emergence of fluoroquinolone resistance, providing critical data for clinicians to guide empirical therapy and adjust topical antibiotic use among ophthalmic patients [37].

SENTRY Antimicrobial Surveillance Program is a global surveillance network that collects clinical isolates from multiple infection sites, including ocular sources in some studies [38]. It monitors resistance trends across over 60 countries, offering insights into geographic variation in multidrug-resistant strains [38]. SENTRY’s ocular-specific data, although less extensive than systemic surveillance, informs clinicians about emerging resistant *Staphylococcus* and *Pseudomonas* strains in the eye [38].

The European Antimicrobial Resistance Surveillance Network (EARS-Net) collects AMR data from European countries, focusing on bloodstream infections and invasive pathogens [39,40,41]. While primarily systemic, the program includes key pathogens implicated in ocular infections, particularly *S. aureus* and *S. pneumoniae* [39,40,41]. EARS-Net provides comparative data across countries, highlighting regional differences in resistance rates that may affect ophthalmic care, especially in surgical prophylaxis and severe keratitis management [39,40,41].

For national and regional AMR surveillance programs, many countries maintain their own surveillance systems, such as the China Antimicrobial Resistance Surveillance System (CARSS), which tracks trends in both Gram-positive and Gram-negative bacteria, including ocular isolates in some hospitals [42,43]. The Australian Group on Antimicrobial Resistance (AGAR) monitors ocular and systemic infections, particularly MRSA and multidrug-resistant Gram-negative organisms [44,45]. The Indian Network for Surveillance of Antimicrobial Resistance (INSAR) provides hospital-based data on ocular and systemic pathogens [46,47]. These national programs complement global efforts, often providing more granular, localized data critical for empiric therapy in ophthalmology. Asia Partnership on Emerging Infectious Diseases Research (APEIRS) in the Asia–Pacific region reveals widespread MDR among Gram-negative isolates, often linked to unregulated antibiotic use [48,49]. In Africa, surveillance remains sparse, but emerging studies suggest an upward trend in resistance across major ocular pathogens [50,51]. Environmental, social, and clinical factors, including frequent empirical use of broad-spectrum antibiotics, suboptimal dosing, poor adherence, and over-the-counter antibiotic availability, accelerate resistance development [52]. Hospital- and community-acquired infections exhibit distinct resistance profiles, with nosocomial infections often demonstrating MDR [53]. Contact lens use, ocular trauma, ocular surface disease, and previous ocular surgery also predispose patients to resistant infections [54,55,56,57]. Anti-microbial resistance in ophthalmology complicates empirical therapy, particularly for sight-threatening infections such as keratitis and endophthalmitis [4]. In areas with high resistance rates, first-line agents such as fluoroquinolones may be inadequate [29], necessitating susceptibility testing and targeted therapy. Multi-drug resistant organisms increase the risk of prolonged infection, surgical intervention, and vision loss [55]. Effective AMS, region-specific guidelines, and routine surveillance are essential to mitigate these risks. Collectively, AMR in ophthalmology is a significant and growing global challenge. Methicillin-resistant *S. aureus*, fluoroquinolone-resistant *P. aeruginosa*, and MDR Gram-negative bacteria represent primary threats to ocular health [12,58]. Patterns of resistance vary by pathogen and geographic region [59], underscoring the importance of local surveillance to inform empirical therapy. Strengthening laboratory capacity, implementing AMS, and monitoring regional trends are critical to preserving the efficacy of available treatments and improving visual outcomes worldwide. Collaborative global surveillance, combined with targeted research, can inform policy, optimize treatment protocols, and reduce the burden of AMR ocular infections (see Table 1).

## 3. Mechanisms of Antimicrobial Resistance in Ocular Pathogens

Antimicrobial resistance in ocular pathogens represents a significant challenge in ophthalmology, complicating the management of infections such as bacterial keratitis, conjunctivitis, and postoperative endophthalmitis [60,61]. The eye’s unique anatomy, immune environment, and frequent exposure to topical antibiotics create a distinct setting for the development and persistence of resistant pathogens [4]. Understanding the mechanisms underlying AMR in ocular pathogens is essential for devising targeted therapies and mitigating the impact of resistance on visual outcomes. The mechanisms can broadly be classified into genetic, biochemical, and structural strategies, including biofilm formation, each contributing to the survival and adaptability of ocular microorganisms in the presence of antimicrobial agents [4,61] as shown in Table 2.

Genetic mechanisms are fundamental drivers of AMR. These mechanisms allow pathogens to acquire and disseminate resistance traits rapidly, enabling adaptation to selective pressures imposed by antibiotic exposure [62]. Mutation-driven resistance occurs when spontaneous genetic mutations alter the target site of an antimicrobial, reducing its binding affinity and efficacy [63]. In ocular pathogens, such mutations are commonly observed in genes encoding bacterial DNA gyrase and topoisomerase IV, conferring resistance to fluoroquinolones, which are frequently used to treat bacterial keratitis and conjunctivitis [64]. Point mutations in the quinolone resistance-determining regions (QRDR) of these enzymes can significantly reduce drug susceptibility, resulting in treatment failures [65,66]. Additionally, mutations in genes encoding penicillin-binding proteins (PBPs) or ribosomal proteins can confer resistance to β-lactams and macrolides, respectively, by altering the drug-binding sites, although such mutations are less frequent in ocular infections [67]. More importantly, Horizontal gene transfer (HGT) is another major genetic mechanism facilitating the spread of resistance among ocular pathogens [68]. The HGT involves the transfer of genetic material between bacteria, often across species boundaries, through mechanisms such as plasmids, transposons, and integrons [68]. Plasmids are extrachromosomal DNA elements capable of carrying multiple resistance genes, including those for β-lactamases, aminoglycoside-modifying enzymes, and macrolide resistance [69]. Transposons, or “jumping genes,” can move resistance determinants between plasmids and chromosomal DNA, further enhancing dissemination [70]. Integrons serve as genetic platforms that capture and express gene cassettes, often encoding resistance to multiple antimicrobial classes [71]. Among these three mobile genetic elements (plasmids, transposons, and integrons), plasmids are considered the most clinically significant drivers of MDR in ocular pathogens [72]. Collectively, these genetic mechanisms enable the rapid acquisition and spread of MDR, particularly in ocular environments where topical or systemic antibiotics are extensively used.

Biochemical mechanisms involve enzymatic or structural modifications that directly impair the activity of antimicrobial agents. These mechanisms are highly diverse and include enzymatic degradation, target modification, efflux pump activation, and reduced membrane permeability [73,74,75]. Enzymatic degradation is a primary strategy employed by many ocular pathogens. β-lactamases, for instance, hydrolyze the β-lactam ring in penicillins, cephalosporins, and related antibiotics, rendering them ineffective [76]. Extended-spectrum β-lactamases (ESBLs) can inactivate a wide range of β-lactams [77], while carbapenemases target carbapenems, often considered last-resort drugs [78]. Aminoglycoside-modifying enzymes, including acetyltransferases, phosphotransferases, and nucleotidyltransferases, chemically modify aminoglycosides such as gentamicin and tobramycin, reducing their binding to bacterial ribosomes and compromising protein synthesis inhibition [69,79]. Target modification represents another biochemical pathway by which pathogens evade antimicrobial action [80]. Fluoroquinolone resistance, for example, is commonly mediated by mutations in DNA gyrase and topoisomerase IV, as mentioned previously [64]. Other target modifications include methylation of ribosomal RNA, which prevents macrolide binding, and alteration of PBPs, which reduces susceptibility to β-lactams [67,81]. These modifications often arise under selective pressure and can be maintained in bacterial populations due to their survival advantage. Efflux pumps are membrane-associated proteins that actively export antibiotics from bacterial cells, lowering intracellular drug concentrations below therapeutic levels [82]. Many ocular pathogens, including *P. aeruginosa* and *S. aureus*, possess multidrug efflux pumps capable of extruding structurally diverse antibiotics, including fluoroquinolones, macrolides, and tetracyclines [83,84]. Efflux-mediated resistance often coexists with other mechanisms, compounding treatment challenges [82]. Reduced permeability of bacterial membranes is another key biochemical mechanism. Alterations in outer membrane porins or lipid composition can limit the penetration of hydrophilic antibiotics such as β-lactams and aminoglycosides [85]. In Gram-negative ocular pathogens like *P. aeruginosa*, loss or modification of porins reduces drug uptake, contributing to MDR [86]. This mechanism is particularly important in ocular infections, where high local concentrations of topical antibiotics may still be insufficient to overcome intrinsic permeability barriers.

Biofilm formation adds another layer of AMR in ocular pathogens, providing a protective environment that facilitates chronic infection and recurrence [4,87]. Biofilms are structured communities of bacteria encased in an extracellular polymeric matrix, which adheres to surfaces such as contact lenses, intraocular lenses, corneal tissue, and ocular prostheses [88]. Within biofilms, bacteria exhibit altered metabolic states and reduced growth rates, making them less susceptible to antibiotics that target actively dividing cells. The extracellular matrix itself can impede drug penetration and sequester antimicrobial agents, further diminishing efficacy [87].

Ocular surface challenges, including tear flow, antimicrobial peptides (AMPs), and immune defenses, influence the establishment and persistence of biofilms [61]. Infections involving biofilm formation, such as chronic bacterial keratitis or device-associated endophthalmitis, often demonstrate resistance that cannot be explained solely by genetic or biochemical mechanisms. These infections may require higher drug concentrations, prolonged therapy, or surgical intervention to achieve resolution [89]. Biofilm-associated resistance also facilitates HGT, as the dense bacterial community allows for close proximity and exchange of plasmids, transposons, and integrons [68]. This synergy between structural, genetic, and biochemical mechanisms underscores the complexity of AMR in ocular pathogens.

The interplay of genetic, biochemical, and biofilm-mediated resistance mechanisms has significant clinical implications. Multi-drug-resistant ocular pathogens compromise the efficacy of empirical therapy, increase the risk of treatment failure, and may necessitate more toxic or costly alternatives. Understanding these mechanisms guides clinicians in selecting appropriate antimicrobial regimens, implementing combination therapies, and considering adjunctive measures such as biofilm disruption or novel drug delivery systems. Additionally, knowledge of resistance mechanisms informs AMS efforts, highlighting the importance of judicious antibiotic use and adherence to evidence-based guidelines. Taken together, AMR in ocular pathogens results from a complex interplay of genetic mutations, HGT, enzymatic degradation, target modification, efflux pumps, reduced permeability, and biofilm formation. These mechanisms enable bacteria to survive antimicrobial therapy, complicating treatment and increasing the risk of vision loss. Understanding these processes is critical for developing effective therapeutics, implementing rapid diagnostic tools, and guiding AMS. Future research should focus on elucidating resistance at the molecular level, innovating drug delivery systems, and disrupting biofilms, equipping ophthalmologists to manage resistant infections and mitigate the growing threat of MDR ocular pathogens.

## 4. Drivers of Resistance in Ophthalmology

While genetic and biochemical mechanisms underpin the ability of pathogens to evade antibiotics, the emergence and spread of resistance are strongly influenced by human and clinical factors. In ophthalmology, several key drivers contribute to AMR, including the overuse and misuse of topical antibiotics, prophylactic use during ocular surgery, subtherapeutic dosing coupled with poor patient compliance, and cross-resistance arising from systemic antibiotic exposure [7,60,90]. Understanding these drivers is crucial for designing stewardship programs, optimizing therapy, and mitigating the growing threat of MDR ocular infections (see Table 3).

One of the most significant contributors to AMR in ophthalmology is the widespread overuse and misuse of topical antibiotics [61]. Topical antimicrobials are commonly prescribed for conditions ranging from mild conjunctivitis to postoperative prophylaxis [91,92]. However, studies have demonstrated that a substantial proportion of these prescriptions are unnecessary or inappropriate [93]. For instance, viral conjunctivitis, which is self-limiting and nonbacterial, is frequently treated with antibiotics due to diagnostic uncertainty or patient demand [94]. Such inappropriate exposure exerts selective pressure on commensal and pathogenic ocular flora, promoting the emergence of resistant strains [94]. The over-the-counter availability of ophthalmic antibiotics in some regions further exacerbates this problem, as patients may self-medicate without clinical guidance, often using incomplete courses or incorrect dosing regimens [95]. Even when prescribed appropriately, repeated or prolonged courses of topical antibiotics increase the risk of selecting for MDR organisms [61]. The cumulative effect of these practices is the amplification of resistant *S. aureus*, *P. aeruginosa*, and *S. pneumoniae* strains on the ocular surface, complicating future infections and limiting therapeutic options.

Prophylactic antibiotics in ocular surgery is a routine practice aimed at preventing postoperative infections such as endophthalmitis [96]. While prophylaxis has contributed to a reduction in infection rates, its widespread and sometimes indiscriminate use has been implicated in driving resistance [97]. Common surgical procedures, including cataract extraction, refractive surgery, and glaucoma interventions, often involve preoperative or postoperative topical antibiotic regimens, sometimes extending over several days or weeks [96]. Extended prophylaxis can expose both ocular and periocular flora to sub-inhibitory concentrations of antibiotics, creating an ideal environment for resistance selection [7]. Furthermore, the choice of broad-spectrum antibiotics for prophylaxis, particularly fluoroquinolones and aminoglycosides, can select MDR organisms on the ocular surface. Studies have shown that repeated surgical procedures and frequent exposure to prophylactic antibiotics are associated with higher colonization rates of MRSA and fluoroquinolone-resistant *Pseudomonas* in patients’ conjunctival flora [98]. This phenomenon not only complicates postoperative infection management but also contributes to the broader community reservoir of resistant pathogens.

Subtherapeutic dosing, whether due to inadequate prescription regimens or poor patient compliance, is another critical driver of AMR in ophthalmology. Topical antibiotics often require frequent administration over several days to achieve effective ocular tissue concentrations. Failure to adhere to these regimens, skipping doses, early discontinuation, or incorrect administration, can result in sub-inhibitory drug levels at the infection site, allowing partially resistant bacteria to survive and multiply [99,100]. Patient-related factors, including difficulty in instilling eye drops, lack of understanding of the regimen, or perceived symptom resolution, frequently contribute to incomplete courses [99,100]. Healthcare provider factors, such as prescribing overly short or overly long courses without clear instructions, also play a role [100]. Subtherapeutic exposure creates selective pressure favoring resistant strains while suppressing susceptible organisms, leading to increased colonization of the ocular surface with resistant bacteria. In addition, insufficient drug penetration in deeper ocular tissues, particularly the cornea or vitreous, can mimic sub-therapeutic exposure, further promoting resistance development in severe infections such as keratitis or endophthalmitis [101].

Cross-resistance between systemic and topical antibiotics represents a less visible but equally important driver of AMR in ophthalmology. Systemic antibiotic use can select for resistant organisms in the ocular surface flora, even in the absence of direct topical exposure. For example, oral fluoroquinolones prescribed for urinary or respiratory infections can select for fluoroquinolone-resistant *Staphylococcus* or *Pseudomonas* strains on the conjunctiva [102]. Similarly, systemic macrolides or β-lactams can influence the susceptibility of ocular *Streptococcus* species [67]. This phenomenon is particularly concerning in regions with high systemic antibiotic consumption, as resistant strains can colonize the eye asymptomatically and later cause difficult-to-treat infections. Cross-resistance also extends to MDR, where exposure to one antimicrobial class confers resistance to structurally unrelated antibiotics via shared mechanisms, such as efflux pumps or plasmid-mediated resistance genes [103]. Consequently, systemic antibiotic stewardship is intrinsically linked to ocular AMR prevention, emphasizing the need for coordinated approaches across medical disciplines [104].

Beyond these primary drivers, several contextual and environmental factors exacerbate AMR in ophthalmology. Hospital and clinic environments, particularly those performing frequent ocular surgeries, can serve as reservoirs for resistant organisms [105]. Contaminated instruments, improper sterilization, or lapses in hand hygiene may facilitate spread [105]. Contact lens use is another contributor; improper hygiene or extended wear can create microenvironments favoring biofilm formation and colonization by resistant *Pseudomonas* and *Staphylococcus* strains [106]. The lack of rapid diagnostics in routine ophthalmic practice further promotes empirical antibiotic use, often resulting in broad-spectrum coverage for suspected infections without definitive pathogen identification or susceptibility testing [107]. This practice amplifies selection pressure and accelerates the emergence of resistance.

The drivers of AMR in ophthalmology have direct implications for patient care. Resistant infections are associated with longer healing times, increased risk of complications such as corneal perforation or vision loss, and higher healthcare costs [6]. Empirical therapy becomes less predictable, necessitating the use of second-line or combination antibiotics, which may have greater toxicity or limited availability [108]. Understanding the underlying drivers, overuse, prophylaxis, subtherapeutic exposure, and cross-resistance, enables ophthalmologists to implement targeted strategies, including judicious prescribing, patient education, adherence monitoring, and coordination with systemic stewardship programs [4,109].

Collectively, the emergence of AMR in ophthalmology is driven by overuse and misuse of topical antibiotics, prophylactic use during ocular surgery, subtherapeutic dosing coupled with poor patient compliance, and cross-resistance with systemic antibiotics. These factors interact with pathogen-specific mechanisms, creating a complex environment that favors the survival and spread of resistant ocular pathogens. Understanding and addressing these drivers is essential for effective infection management, stewardship implementation, and the preservation of visual outcomes. Future interventions should integrate patient education, evidence-based prescribing, rapid diagnostics, and coordination with systemic stewardship efforts to curb the growing threat of resistant ocular infections.

## 5. Evolution of Multidrug-Resistant Ocular Pathogens

Historically, bacterial infections of the eye, including conjunctivitis, keratitis, and endophthalmitis, have been effectively managed with topical and systemic antibiotics. However, the emergence and proliferation of MDR ocular pathogens now present a substantial challenge to both clinicians and patients. Understanding the evolutionary drivers of resistance, documenting illustrative cases, and assessing the clinical and economic impact of resistant infections are critical steps in formulating effective management strategies.

The emergence of MDR pathogens in ophthalmology is primarily driven by selective pressure from repeated or inappropriate antibiotic exposure [6]. Topical antibiotics, which are widely prescribed for ocular surface infections and prophylaxis in ophthalmic surgery, often achieve subtherapeutic concentrations in the corneal tissue [110]. Such sub-lethal antibiotic exposure creates an environment conducive to the selection of resistant strains [111]. The most commonly implicated organisms include *S. aureus*, *S. pneumoniae*, *P. aeruginosa*, and other Gram-negative bacilli [4]. Methicillin-resistant *S. aureus* is of particular concern in ocular infections, with studies demonstrating its increasing prevalence in both community-acquired and nosocomial cases of conjunctivitis, keratitis, and postoperative endophthalmitis [112]. Methicillin-resistant *S. aureus* ocular infections are often associated with higher rates of treatment failure, prolonged healing times, and increased risk of vision-threatening complications [113].

*Pseudomonas aeruginosa*, particularly virulent in contact lens-associated keratitis, is notorious for its intrinsic resistance mechanisms, including efflux pumps, enzymatic degradation of antibiotics, and biofilm formation [83,106]. The MDR strains of *P. aeruginosa* often exhibit resistance to fluoroquinolones, aminoglycosides, and cephalosporins, which complicates empirical therapy and necessitates the use of combination regimens or newer, less accessible antibiotics [29,114,115]. Similarly, *S. pneumoniae*, traditionally susceptible to penicillin and macrolides, has seen a rising prevalence of resistant strains, particularly in the context of ocular surface infections in children and the elderly [116,117]. The global dissemination of penicillin-non-susceptible and macrolide-resistant strains represents a major clinical challenge, given the limited number of topical and systemic agents effective against these organisms [118].

Several clinical studies highlight the severity of MDR ocular infections. For example, a retrospective analysis of bacterial keratitis in a tertiary care center reported that MRSA accounted for 15–20% of cases, with affected patients requiring extended courses of fortified topical vancomycin and, in some cases, surgical intervention due to corneal perforation [119,120]. In comparison, MRSA infections responded well to conventional first-line antibiotics, illustrating the profound impact of resistance on therapeutic efficacy [121]. Similarly, *P. aeruginosa* keratitis remains a significant cause of corneal morbidity worldwide [83,106]. Cases involving MDR strains frequently present with rapid corneal melting, hypopyon formation, and, in severe instances, endophthalmitis [122,123]. These infections often require aggressive, hospital-based management, including hourly administration of fortified antibiotics and, in refractory cases, surgical debridement or keratoplasty [124]. The mortality rate for ocular *Pseudomonas* infections is negligible; however, the morbidity, particularly permanent visual loss, is substantial [125].

Antimicrobial resistance directly affects clinical outcomes in ophthalmology. Infections with resistant organisms are associated with prolonged disease courses, increased rates of complications such as corneal perforation or retinal involvement, and higher likelihood of surgical intervention [4]. For instance, MRSA keratitis has been shown to double the median healing time compared to methicillin-susceptible infections [126], and MDR *Pseudomonas* keratitis carries a higher risk of requiring therapeutic keratoplasty [127].

Rising resistance also undermines empirical therapy, which traditionally relies on broad-spectrum antibiotics such as fluoroquinolones [6], increasing reliance on culture and sensitivity testing to guide targeted therapy. While essential, this delays targeted therapy and potentially worsens outcomes [128]. Furthermore, treatment failures can contribute to recurrent infections, promoting further resistance development and perpetuating a cycle that is difficult to break [9,129]. In ophthalmology, the stakes are high because ocular infections can directly threaten vision. Corneal infections caused by resistant organisms can rapidly progress to corneal ulcers, perforations, or scarring, all of which compromises visual acuity [130]. Endophthalmitis caused by resistant pathogens often leads to irreversible vision loss despite aggressive medical and surgical management [131]. The need for early, appropriate intervention is critical, but resistance limits the available effective agents, leaving clinicians with few options that may be toxic or less accessible.

Beyond clinical implications, resistant ocular infections carry substantial economic cost. Patients with MDR infections often require prolonged hospital stays, frequent follow-up visits, and intensive treatment regimens that include fortified antibiotics, intravitreal injections, or surgical procedures. The cumulative cost of managing these cases is significantly higher than treating susceptible infections. Moreover, the indirect costs, such as loss of productivity due to visual impairment or hospitalization, further compound the economic impact. Healthcare systems are also strained by the need for ongoing surveillance, microbiological testing, and the development of antimicrobial stewardship programs specifically tailored for ophthalmology. Inadequate stewardship can accelerate resistance trends, potentially leading to a scenario in which routine infections become increasingly difficult or impossible to manage effectively (see Table 4).

## 6. Diagnostic Advances in Ophthalmology

The field of ophthalmology has witnessed remarkable diagnostic advancements in recent years, particularly in the detection of AMR and ocular pathogens. These developments have been driven by the increasing prevalence of drug-resistant infections, the urgent need for timely pathogen identification, and the demand for point-of-care (POC) solutions that enable immediate clinical decision-making. Modern diagnostic tools now integrate molecular techniques, rapid resistance detection assays, and portable POC platforms, collectively improving patient outcomes, optimizing antimicrobial therapy, and enhancing public health surveillance (see Table 5).

Traditional culture-based methods, while reliable, often require 48–72 h or longer to yield results. During this period, empirical therapy may be ineffective, leading to prolonged infection, tissue damage, or irreversible vision loss. Rapid diagnostics overcome this limitation by providing clinicians with actionable results within hours, enabling precise, targeted interventions [132]. Key pathogens targeted include MRSA, MDR *P. aeruginosa*, vancomycin-resistant *Enterococcus* spp., and drug-resistant *S. pneumoniae* [132].

Molecular diagnostics have revolutionized ocular pathogen identification by offering high sensitivity, specificity, and rapid turnaround times [133]. Polymerase Chain Reaction (PCR), including multiplex and broad-range PCR, allows simultaneous detection of multiple pathogens from a single ocular sample [134]. Polymerase Chain Reaction is particularly valuable in conditions such as infectious uveitis, keratitis, and endophthalmitis, where early identification of the causative agent is critical for preserving vision [134]. Unlike culture-based methods, PCR can detect both viable and non-viable organisms, which is especially important when prior antibiotic therapy may suppress microbial growth [135]. Metagenomic next-generation sequencing (mNGS) represents an unbiased, comprehensive approach to pathogen detection [136]. By sequencing all nucleic acids in a sample, mNGS can identify bacteria, viruses, fungi, and parasites without prior knowledge of the suspected pathogen [136]. This approach has been instrumental in diagnosing culture-negative endophthalmitis, rare ocular infections, and emerging pathogens [137]. Additionally, mNGS can simultaneously detect resistance genes, providing clinicians with actionable information to guide antimicrobial therapy [138].

Targeted molecular assays are also emerging. One example is the NanoString nCounter SPRINT Profiler, which utilizes multiplexed panels to simultaneously detect 46 ocular pathogens and multiple resistance or virulence markers [139,140]. This approach significantly increases diagnostic yield and is particularly useful in complex infections such as endophthalmitis, keratitis, and post-surgical ocular infections [139,140]. Similarly, the MTBDRplus assay targets intraocular tuberculosis and detects *Mycobacterium tuberculosis* (*M. tuberculosis*) along with drug resistance determinants [141,142]. Given the slow growth of *M. tuberculosis* in culture, molecular assays like MTBDRplus drastically reduce diagnostic time, improving clinical outcomes and guiding therapy [141,142].

For MDR Gram-negative organisms, the RESIST ACINETO rapid test enables simultaneous detection of four key resistance determinants in *Acinetobacter baumannii*, a pathogen increasingly implicated in hospital-acquired ocular infections [143,144]. By rapidly identifying carbapenem-resistant strains, clinicians can promptly modify therapy, avoid ineffective antibiotics and prevent further resistance development [143,144]. Other emerging rapid resistance detection methods include fluorescence-based susceptibility testing and microfluidic platforms that detect metabolic changes in pathogens exposed to antibiotics [145]. These technologies provide near real-time information on susceptibility profiles and can be adapted for high-throughput screening in tertiary care ophthalmology centers [145].

Nanopore targeted sequencing (NTS) is another innovative molecular tool that allows real-time, rapid identification of ocular pathogens [146,147]. The NTS can sequence DNA directly from clinical specimens, offering results within hours [146,147]. This capability is particularly valuable in acute cases of endophthalmitis or keratitis, where delayed treatment may result in permanent visual impairment [146,147]. Beyond identification, NTS can provide insight into microbial genetic variations, virulence factors, and resistance mechanisms, supporting precision medicine approaches in ophthalmology [146,147].

Point-of-care testing has emerged as a pivotal component in modern ophthalmic diagnostics, offering rapid results directly at the site of care [148]. This is particularly advantageous in emergency situations, outpatient clinics, and resource-limited settings, where timely laboratory access may be limited [149]. Point-of-care testing reduces turnaround time, facilitates immediate clinical decisions, and improves patient compliance [150]. Among current POC technologies, lateral flow assays (LFAs) stand out for their simplicity and speed. For example, the *Aspergillus*-specific lateral flow device (AspLFD) enables rapid detection of fungal pathogens in microbial keratitis and endophthalmitis [151]. LFAs offer a cost-effective diagnostic solution with high sensitivity and specificity while requiring minimal operator training, making them suitable for both tertiary hospitals and rural clinics [152]. Advances in isothermal amplification methods have further expanded POC capabilities. Recombinase polymerase amplification (RPA) allows rapid nucleic acid amplification at a constant temperature, eliminating the need for thermal cycling as required by PCR [153]. Unlike PCR, RPA does not require thermal cycling equipment, allowing rapid amplification of nucleic acids at a constant temperature [153]. Results can be obtained in as little as 20–30 min, facilitating immediate treatment decisions [154]. Similarly, cycling probe technology (CPT) provides linear amplification of target DNA sequences at a single temperature, offering a sensitive and portable approach to ocular pathogen detection [155]. Recent advancements have integrated machine learning (ML) algorithms into POC platforms. These intelligent systems can analyze complex data patterns from ocular samples, such as nucleic acid amplification curves or metabolic changes in microbial cultures, enhancing diagnostic accuracy and enabling automated interpretation [156]. ML-enhanced devices reduce human error, standardize results across settings, and may ultimately predict resistance trends based on local epidemiology and patient-specific factors [156]. The combination of molecular methods with POC testing has led to the development of integrated diagnostic platforms capable of pathogen identification, resistance profiling, and therapeutic guidance in a single device. These platforms streamline workflow, reduce the need for multiple laboratory tests, and allow clinicians to initiate targeted therapy within hours, rather than days.

The integration of rapid resistance detection, molecular diagnostics, and POC testing has profound clinical implications. Early and accurate identification of ocular pathogens significantly reduces the risk of vision loss and complications associated with delayed or inappropriate therapy. Rapid diagnostics also support AMS by minimizing empiric use of broad-spectrum antibiotics, thereby reducing the emergence of MDR strains [60]. From a public health perspective, these tools facilitate epidemiological surveillance, enabling early detection of resistant outbreaks and informing local, regional, and global treatment guidelines [157]. Molecular and rapid POC diagnostics can also detect asymptomatic carriers of resistant organisms, preventing the spread of pathogens within healthcare facilities and communities [157]. Furthermore, real-time surveillance data generated from these technologies can guide public health policy, infection control strategies, and AMS initiatives.

The future of ophthalmic diagnostics is poised for continued innovation. Advances in microfluidics, nanotechnology, and biosensor integration are expected to produce miniaturized, fully automated devices capable of detecting multiple pathogens and resistance genes from minute ocular samples [158]. Integration with digital health platforms, teleophthalmology, and cloud-based data systems could enable remote diagnostics, real-time consultation, and centralized surveillance, expanding access to high-quality care even in underserved regions [159,160]. Additionally, AI and predictive analytics may enable personalized therapy, predicting the likelihood of resistance based on microbial genomic data, local epidemiology, and patient history [161]. Such technologies could revolutionize clinical decision-making, ensuring each patient receives the most effective, targeted therapy while preserving vision and minimizing unnecessary antibiotic exposure [161].

## 7. Strategies to Mitigate Antimicrobial Resistance in Ophthalmology

To combat this escalating problem, multifaceted strategies are essential, encompassing AMS, innovative drug delivery approaches, non-antibiotic therapies, and preventive interventions such as vaccination and host-directed therapies.

Antimicrobial stewardship refers to a coordinated set of strategies aimed at optimizing the use of antimicrobial agents to improve patient outcomes while minimizing resistance [162]. In ophthalmology, AMS is crucial due to the frequent empirical use of topical antibiotics for conditions such as bacterial conjunctivitis, keratitis, and post-surgical prophylaxis. Inappropriate prescribing, prolonged use, or sub-therapeutic dosing fosters resistant strains such as MRSA and multidrug-resistant *P. aeruginosa* [163]. Implementing stewardship programs in ophthalmology involves several key steps. First, guidelines based on local resistance patterns should guide empirical therapy. For instance, studies have demonstrated variability in MRSA prevalence between regions, making local antibiograms a vital tool for appropriate antibiotic selection. Second, education of ophthalmologists, nurses, and allied health personnel on judicious antibiotic use helps reduce unnecessary prescriptions. Third, diagnostic precision must be enhanced. Rapid diagnostic tests, including PCR-based detection and culture-guided therapy, can ensure that antibiotics are reserved for confirmed bacterial infections rather than viral or allergic conditions. Finally, audit and feedback mechanisms, where prescribing patterns are monitored and clinicians receive feedback, have been shown to improve compliance with stewardship principles [164,165,166].

Traditional topical eye drops face limitations such as rapid tear turnover, poor ocular surface penetration, and low patient compliance [167]. Novel drug delivery systems are emerging as solutions to these challenges. Nanoparticles have garnered attention due to their ability to encapsulate antibiotics, enhance penetration into ocular tissues, and provide sustained release [168]. For example, polymeric nanoparticles loaded with fluoroquinolones or aminoglycosides can increase bioavailability at the site of infection while reducing dosing frequency [169,170]. Similarly, liposomal formulations and hydrogels allow for prolonged ocular surface residence time, enhancing drug efficacy and potentially reducing the selection pressure for resistant bacteria [171]. Sustained-release implants, such as intraocular inserts or subconjunctival depots, offer another promising approach [172]. These systems can deliver consistent therapeutic levels over days to weeks, minimizing the peaks and troughs associated with conventional topical therapy. By maintaining optimal drug concentrations, sustained-release formulations reduce the likelihood of sublethal exposure that drives resistance [169,170]. Preclinical studies have demonstrated the efficacy of such implants in bacterial keratitis models, and clinical trials are underway to evaluate their safety in human patients [173].

Given the limitations of antibiotics and the rising prevalence of resistant strains, non-antibiotic therapies are gaining traction in ophthalmology. Phage therapy, the use of bacteriophages to target specific bacterial pathogens, has shown promise in treating multidrug-resistant ocular infections [19]. Phages are highly specific, reducing the impact on commensal flora and minimizing the risk of resistance development in non-target bacteria [174]. In vitro and animal studies have demonstrated the efficacy of phage therapy against *P. aeruginosa* and *S. aureus* keratitis, with ongoing clinical research exploring its safety and potential integration into ophthalmic practice [175,176,177]. Antimicrobial peptides represent another innovative approach [178]. These naturally occurring molecules, part of the innate immune system, exhibit broad-spectrum antimicrobial activity by disrupting bacterial membranes and interfering with intracellular processes. Synthetic or recombinant AMPs have been investigated as topical formulations for ocular infections, offering rapid bactericidal action and a low propensity for resistance development [178]. Challenges remain in terms of formulation stability and ocular tolerance, but early studies are encouraging [178].

Preventing infections in the first place is arguably the most effective strategy against AMR. Vaccination against common ocular pathogens, while still in the early stages of development, could significantly reduce the incidence of bacterial keratitis and conjunctivitis. For example, vaccines targeting *S. pneumoniae* and *Haemophilus influenzae* have the potential to lower ocular infections, particularly in pediatric populations where conjunctivitis is prevalent [179]. Reducing infection rates naturally decreases antibiotic use and, consequently, selection pressure for resistant strains. Host-directed therapies represent another preventive and therapeutic frontier [180]. These interventions enhance the host’s immune response rather than directly targeting pathogens, thereby reducing the reliance on antibiotics [180,181]. Approaches include the use of immunomodulatory agents, anti-inflammatory peptides, and small molecules that boost innate immunity on the ocular surface [182,183]. The topical formulations that increase the expression of AMPs in corneal epithelial cells have been shown to accelerate bacterial clearance without promoting resistance [184].

Antimicrobial resistance in ophthalmology poses a critical and escalating threat to the success of current therapeutic strategies and long-term visual outcomes. Addressing this challenge demands an integrated, evidence-based framework that combines antimicrobial stewardship, innovative drug delivery platforms, and the exploration of non-antibiotic modalities such as bacteriophage therapy and immunomodulation. Preventive strategies, including vaccination, improved infection control, and host-targeted interventions, play a complementary role in reducing pathogen transmission and disease incidence (see Table 6). A concerted global effort encompassing clinical practice, research innovation, and public health policy is essential to preserve antibiotic efficacy, protect vision, and sustain the future of ophthalmic care.

## 8. Future Directions

Integration of genomic surveillance with existing antimicrobial stewardship programs offers a promising strategy to reduce inappropriate antibiotic use and curb the emergence of MDR ocular infections. By linking pathogen genomics with local resistance data, clinicians can anticipate resistance trends and adjust therapy more precisely. Complementing this, AI and ML can leverage large-scale clinical, microbiological, and genomic datasets to predict which patients are at higher risk of treatment failure. For instance, AI-driven analysis of electronic health records and laboratory susceptibility results could guide early, targeted interventions in conditions such as bacterial keratitis, endophthalmitis, and postoperative infections, reducing morbidity and healthcare costs. This approach is supported by evidence that AI and digital health technologies can accelerate drug discovery, optimize lead compounds, and support personalized treatment using multi-modal biomarkers, ultimately shortening development timelines and enhancing success rates [185].

Personalized antimicrobial therapy represents a transformative approach by tailoring treatments to both the patient and the pathogen. Combining genomic information, local resistance patterns, and host-specific factors, including immune status and ocular surface microbiome composition, enables clinicians to select the most effective agent at the optimal dose. This strategy minimizes subtherapeutic exposure, preserves normal ocular microbiota, and reduces selective pressure driving resistance. Rapid molecular assays or point-of-care sequencing could further facilitate real-time, patient-specific decision-making in ophthalmology. For example, cationic peptides such as melimine have been incorporated into silicone hydrogel contact lenses and shown in preclinical models to prevent bacterial keratitis and contact lens–associated ocular complications by directly reducing pathogen colonization [186].

Alternative therapeutics, including bacteriophage therapy, illustrate the potential of precision-guided interventions. In preclinical studies, six anti–*S. aureus* phages were tested against 50 MDR ocular MRSA isolates, with select phages demonstrating broad activity across both MDR and community-associated lineages, highlighting the feasibility of phage-based treatments [187]. Similarly, an in situ gel-forming eye drop containing a Cystoviridae bacteriophage against *P. aeruginosa* showed effective corneal re-epithelialization and restoration of normal tissue architecture in animal models, indicating a promising therapeutic avenue for *P. aeruginosa* keratoconjunctivitis [188]. These examples underscore the value of combining novel antimicrobial strategies with data-driven precision medicine to target resistant ocular pathogens effectively.

Finally, global collaboration remains crucial to addressing AMR comprehensively. Resistant ocular pathogens do not respect national borders, and inconsistent surveillance or stewardship practices can undermine local efforts. International networks enabling standardized data sharing, harmonized reporting, and coordinated clinical trials will be essential for generating robust evidence on effective interventions. Collaborative research also accelerates the development of innovative therapeutics, including bacteriophages, AMPs, and vaccines, while fostering responsible antimicrobial stewardship. The future of combating AMR in ophthalmology lies at the intersection of genomics, AI, precision medicine, and international cooperation. By leveraging these innovations, clinicians can anticipate resistance patterns, personalize therapy, and preserve the efficacy of existing antimicrobials, ultimately safeguarding vision and public health worldwide.

## 9. Conclusions

**Figure 1:**
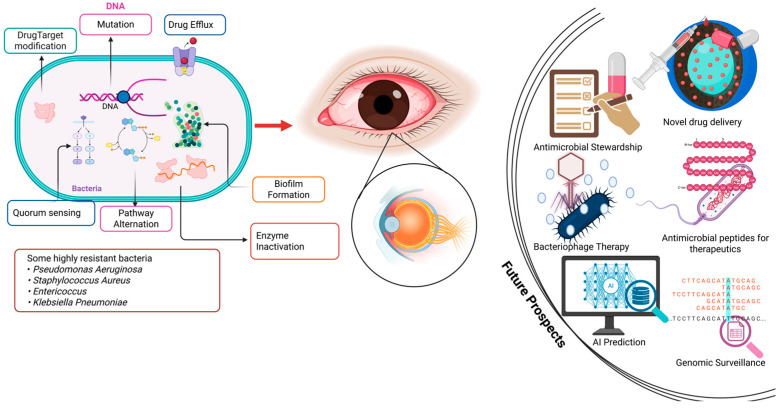
Mechanisms of AMR and future therapeutic strategies. Images was developed in Biorender.com by I.O.D.J.

Antimicrobial resistance in ophthalmology arises from a combination of microbial adaptation and selective pressure from antibiotic exposure. Mechanisms include enzymatic drug inactivation, efflux pumps, target site modifications, and biofilm formation, all of which enable ocular pathogens such as *S. aureus*, *P. aeruginosa*, and *S. pneumoniae* to survive conventional therapies (see Figure 1). Over time, these mechanisms have evolved under pressures from overuse, prophylactic misuse, and subtherapeutic dosing, leading to the emergence of multidrug-resistant strains that challenge standard treatment paradigms. Clinically, AMR complicates the management of bacterial keratitis, conjunctivitis, and postoperative infections, increasing the risk of treatment failure, prolonged inflammation, vision loss, and healthcare costs. Resistant infections often require broader-spectrum or combination therapies, which may carry higher toxicity and further drive resistance. Addressing AMR in ophthalmology requires an integrated approach combining antimicrobial stewardship, rapid diagnostics, and innovative research. Stewardship programs must promote rational prescribing, while molecular and genomic tools can guide targeted therapy. Simultaneously, ongoing research into novel antimicrobials, alternative therapies, and predictive AI models is critical to anticipate resistance trends. Only through coordinated clinical vigilance and scientific innovation can the field preserve the efficacy of existing treatments and protect visual health globally.

## Figures and Tables

**Table 1:** AMR surveillance networks relevant to ocular pathogens.

Surveillance Program	Scope/Focus	Pathogens	Notes
GLASS (WHO)	Global AMR surveillance	*S. aureus*, *P. aeruginosa*, *S. pneumoniae*	Primarily systemic infections, but data extrapolated to ophthalmology
ARMOR (US)	Ocular-specific	MRSA, fluoroquinolone-resistant *P. aeruginosa*, penicillin-resistant *S. pneumoniae*	Tracks conjunctivitis, keratitis, endophthalmitis; informs empiric therapy
Ocular TRUST (US)	Ocular-specific	*S. aureus*, *S. pneumoniae*, *H. influenzae*, *P. aeruginosa*	Documents MRSA and fluoroquinolone resistance trends
SENTRY (Global)	Multi-site, multi-country	*S. aureus*, *P. aeruginosa*	Includes ocular isolates in some studies; highlights geographic resistance variation
EARS-Net (Europe)	Systemic, invasive infections	*S. aureus*, *S. pneumoniae*	Provides regional comparative data affecting ophthalmic care
CARSS (China)	National AMR surveillance	Gram-positive and Gram-negative bacteria	Some hospitals include ocular isolates
AGAR (Australia)	National AMR surveillance	MRSA, MDR Gram-negative organisms	Covers ocular and systemic infections
INSAR (India)	National hospital-based surveillance	Ocular and systemic pathogens	Provides local data for empiric therapy
APEIRS (Asia-Pacific)	Regional surveillance	MDR Gram-negative isolates	Highlights impact of unregulated antibiotic use

AGAR, Australian Group on Antimicrobial Resistance; APEIRS, Asia-Pacific Emerging Infections Network; ARMOR, Antimicrobial Resistance Monitoring in Ocular MicRoorganisms; CARSS, China Antimicrobial Resistance Surveillance System; EARS-Net, European Antimicrobial Resistance Surveillance Network; GLASS, Global Antimicrobial Resistance and Use Surveillance System; INSAR, Indian Society for Clinical Research; Ocular TRUST, Ocular Tracking Resistance in the U.S. Today; SENTRY, SENTRY Antimicrobial Surveillance Program.

**Table 2:** Mechanisms of AMR in ocular pathogens and their clinical implications.

Mechanism	Description	Examples	Clinical Implications
Genetic Mechanisms	Acquisition or alteration of genetic material that confers resistance. Includes mutation-driven resistance and HGT.	-Mutations in DNA gyrase/topoisomerase IV → fluoroquinolone resistance -Mutations in PBPs or ribosomal proteins → β-lactam or macrolide resistance (less frequent) -HGT via plasmids, transposons, integrons carrying resistance genes	Rapid acquisition of MDR, treatment failure, spread of resistance across species
Biochemical Mechanisms	Alterations that directly impair antimicrobial activity. Includes enzymatic degradation, target modification, efflux pumps, and reduced permeability.	-β-lactamases, ESBLs, carbapenemases → β-lactam inactivation -Aminoglycoside-modifying enzymes → gentamicin/tobramycin resistance -Target modifications (e.g., ribosomal RNA methylation, PBP changes) -Multidrug efflux pumps (fluoroquinolones, macrolides, tetracyclines) -Reduced outer membrane porin permeability	Reduced drug efficacy, multidrug resistance, may require higher or combination therapies
Biofilm Formation	Structured bacterial communities encased in extracellular polymeric matrix that adheres to surfaces. Provides physical protection and metabolic dormancy.	-Biofilms on contact lenses, intraocular lenses, corneal tissue, ocular prostheses	Chronic/recurrent infections, reduced antibiotic penetration, facilitation of HGT, may necessitate prolonged therapy or surgical intervention
Interplay of Mechanisms	Synergistic effects of genetic, biochemical, and biofilm-mediated resistance.	-MDR pathogens with plasmid-borne genes inside biofilms expressing efflux pumps	Higher risk of treatment failure, vision loss, need for advanced therapeutics and AMS

AMS, Antimicrobial Stewardship; HGT, Horizontal Gene Transfer; MDR, Multi-drug resistance; PBP, penicillin-binding proteins.

**Table 3:** Drivers of AMR in ophthalmology.

Driver	Description	Examples	Clinical Implications
Overuse and misuse of topical antibiotics	Frequent, unnecessary, or inappropriate prescriptions increase selective pressure on ocular flora	-Antibiotics used for viral conjunctivitis -Over-the-counter self-medication -Prolonged or repeated courses	-Emergence of resistant *S. aureus*, *P. aeruginosa*, *S. pneumoniae* -Complicated future infections -Limited therapeutic options
Prophylactic antibiotics in ocular surgery	Routine pre- or postoperative use exposes ocular flora to sub-inhibitory drug concentrations	-Cataract, refractive surgeries, glaucoma surgeries -Use of broad-spectrum antibiotics (fluoroquinolones, aminoglycosides)	-Selection for multidrug-resistant organisms (MRSA, fluoroquinolone-resistant *Pseudomonas*) -Increased postoperative infection risk -Contribution to community resistance reservoir
Subtherapeutic dosing/poor patient compliance	Inadequate dosing or incorrect administration reduces antibiotic effectiveness	-Skipped doses, early discontinuation -Difficulty instilling drops -Prescribing errors	-Partial survival of resistant bacteria -Increased ocular colonization with resistant strains -Deeper tissue infections (keratitis, endophthalmitis) are more difficult to treat
Cross-resistance from systemic antibiotics	Systemic antibiotic use selects for resistant ocular flora even without topical exposure	-Oral fluoroquinolones → fluoroquinolone-resistant *Staphylococcus* or *Pseudomonas* -Macrolides or β-lactams affecting ocular *Streptococcus* spp.	-Asymptomatic colonization leading to hard-to-treat infections -Development of MDR via shared mechanisms (e.g., efflux pumps, plasmids)
Environmental/contextual factors	External conditions that facilitate resistance spread	-Hospital/clinic reservoirs (contaminated instruments, poor hygiene) -Contact lens misuse (biofilm formation) -Lack of rapid diagnostics → empirical broad-spectrum use	-Increased transmission of resistant strains -Amplified selection pressure -Empiric therapy is less predictable, higher risk of complications
Consequences for patient care	Result of the combined drivers above	-Longer healing times -Risk of corneal perforation or vision loss -Need for second-line or combination antibiotics	-Greater toxicity or limited availability of alternatives -Increased healthcare costs -Reduced efficacy of empiric therapy

**Table 4:** Evolution and impact of MDR ocular pathogens.

Category	Examples	Clinical Implications	Economic/Healthcare Impact	Evolutionary Perspective
Drivers of Resistance	-Repeated or inappropriate antibiotic use -Subtherapeutic corneal drug levels from topical antibiotics -Contact lens use and nosocomial exposure	-Selection of resistant strains -Increased prevalence of MDR pathogens	-Necessitates ongoing surveillance and stewardship programs	-Sub-lethal antibiotic exposure favors survival of resistant mutants -HGT and selection pressure accelerate emergence of MDR strains
Key MDR Pathogens	-*S. aureus* (MRSA) -*P. aeruginosa* (MDR strains) -*S. pneumoniae* (penicillin- or macrolide-resistant) -Other Gram-negative bacilli	-MRSA: treatment failure, prolonged healing -*P. aeruginosa*: rapid corneal melting, hypopyon, endophthalmitis -*S. pneumoniae*: limited effective antibiotics	-Limited therapeutic options increase treatment complexity and cost	-Resistant strains often arise from point mutations, acquisition of resistance genes, or selective survival in hospital/community settings
Clinical Manifestations	-Conjunctivitis, keratitis, endophthalmitis -Corneal ulceration, perforation, scarring -Rapid progression in contact lens-associated infections	-Prolonged disease courses -Higher likelihood of surgical intervention (keratoplasty, debridement) -Risk of permanent vision loss	-Increased hospital stays -Intensive treatments required (fortified antibiotics, intravitreal injections, surgery)	-MDR pathogens can dominate ocular microbiota over time, leading to recurring and harder-to-treat infections
Impact on Treatment	-Reduced efficacy of first-line broad-spectrum antibiotics (e.g., fluoroquinolones) -Need for culture and sensitivity-guided therapy	-Delayed targeted therapy -Recurrent infections and perpetuation of resistance cycle	-Longer treatment duration and monitoring -Increased burden on healthcare resources	-Continuous antibiotic pressure drives adaptive mutations and selection of MDR clones
Economic Burden	-Direct: prolonged hospitalization, multiple follow-ups, intensive therapy -Indirect: loss of productivity due to visual impairment	-Higher cumulative cost per patient -Increased strain on healthcare systems	-Resource-intensive management -Need for specialized AMS	-Persistent MDR infections increase systemic healthcare costs and drive demand for novel antibiotics

AMS, Antimicrobial Stewardship; HGT, Horizontal Gene Transfer; MDR, Multidrug resistance; MRSA, methicillin-resistant *S. aureus*.

**Table 5:** Diagnostic strategies for MDR ocular pathogens.

Diagnostic Group	Techniques/Tools	Key Features	Clinical and Public Health Implications
Traditional Culture-Based Methods	-Standard microbiological culture -Susceptibility testing	-48–72 h turnaround -Identifies viable organisms	-Reliable but slow -Empiric therapy may be ineffective during delay
Molecular Diagnostics	-PCR (multiplex, broad-range) -mNGS -Targeted assays (NanoString nCounter, MTBDRplus)	-High sensitivity and specificity -Detects multiple pathogens simultaneously -Identifies resistance genes -Works on viable and non-viable organisms	-Early, accurate pathogen ID -Guides targeted therapy -Reduces inappropriate antibiotic use -Useful in culture-negative infections
Rapid Resistance Detection Methods	-RESIST ACINETO (Acinetobacter) -Fluorescence-based susceptibility testing -Microfluidic platforms	-Detect metabolic or genetic resistance determinants quickly -Provides near real-time susceptibility profiles	-Optimizes therapy -Avoids ineffective antibiotics -Prevents further resistance development
Point-of-Care (POC) Testing	-Lateral flow assays (AspLFD) -RPA -CPT -ML-enhanced POC platforms	-Rapid results (minutes to <1 h) -Portable and simple to use -Suitable for resource-limited settings	-Immediate clinical decisions -Reduces delays in treatment -Enhances diagnostic access and compliance
Innovative and Next-Generation Diagnostics	-NTS -Microfluidics + Biosensors -AI and predictive analytics -Teleophthalmology and cloud-based integration	-Real-time sequencing and resistance profiling -Miniaturized, fully automated systems -Remote diagnostics possible	-Enables precision medicine -Improves epidemiological surveillance -Supports AMS and personalized therapy

CPT, Cycling Probe Technology; mNGS, Metagenomic Next-Generation Sequencing; NTS, Nanopore Targeted Sequencing; PCR, Polymerase Chain Reaction; POC, Point-of-Care; RPA, Recombinase Polymerase Amplification.

**Table 6:** Strategies to mitigate AMR in ophthalmology.

Strategy	Examples	Mechanism	Clinical Implications
Antimicrobial Stewardship (AMS)	-Guidelines based on local resistance patterns -Education of ophthalmologists, nurses, and allied health personnel -Rapid diagnostics (PCR, culture-guided therapy) -Audit and feedback on prescribing patterns	Optimize antibiotic use, reduce unnecessary prescriptions, minimize selection of resistant strains	-Reduces incidence of MDR infections -Improves treatment outcomes -Limits perpetuation of resistance cycles
Novel Drug Delivery Systems	-Nanoparticles (polymeric, liposomal) loaded with antibiotics -Hydrogels for sustained release -Intraocular or subconjunctival sustained-release implants	Enhance ocular penetration, prolong drug residence time, maintain therapeutic concentrations, reduce sublethal exposure	-Improved drug efficacy -Reduced dosing frequency -Lower risk of developing resistance
Non-Antibiotic Therapies	-Phage therapy targeting specific pathogens (e.g., *P. aeruginosa*, *S. aureus*) -AMPs for rapid bactericidal action	Directly kill or inhibit bacteria without using conventional antibiotics, reducing selective pressure	-Potential treatment for MDR infections -Preserves commensal flora -Low propensity for resistance development
Preventive Interventions	-Vaccination against *S. pneumoniae*, *Haemophilus influenzae* -Host-directed therapies (immunomodulatory agents, peptides enhancing innate immunity)	Reduce infection incidence and boost host defenses	-Decreased antibiotic use -Reduced selection pressure for resistance -Lower overall burden of ocular infections

## Data Availability

The data supporting the conclusion of this paper is enclosed in the manuscript.

## Abbreviations

AGAR: Australian Group on Antimicrobial Resistance; AI: Artificial intelligence; AMP: Antimicrobial peptide; AMR: Antimicrobial resistance; APEIRS: Asia Partnership on Emerging Infectious Diseases Research; AMS: Antimicrobial stewardship; ARMOR: Antibiotic Resistance Monitoring in Ocular Microorganisms; AspLFD: Aspergillus-specific lateral-flow device; CARSS: China Antimicrobial Resistance Surveillance System; CoNS: coagulase-negative staphylococci; CPT: Cycling probe technology; EARS-Net: European Antimicrobial Resistance Surveillance Network; ESBL: Extended-spectrum β-lactamase; GLASS: Global Antimicrobial Resistance and Use Surveillance System; HGT: Horizontal gene transfer; INSAR: Indian Network for Surveillance of Antimicrobial Resistance; IOTB: Intraocular tuberculosis; LFA: Lateral flow assay; MDR: Multidrug-resistant; ML: Machine learning; mNGS: Metagenomic next-generation sequencing; MRSA: methicillin-resistant Staphylococcus aureus; NTS: Nanopore targeted sequencing; PBP: Penicillin-binding protein; POC: point-of-care; PCR: Polymerase Chain Reaction; RPA: Recombinase polymerase amplification; TRUST: Tracking Resistance in the United States Today; QRDR: Quinolone resistance-determining regions; WHO: World Health Organization.
